# Towards liver segmentation in the wild via contrastive distillation

**DOI:** 10.1007/s11548-023-02912-3

**Published:** 2023-05-05

**Authors:** Stefano Fogarollo, Reto Bale, Matthias Harders

**Affiliations:** 1 Department of Computer Science Interactive Graphics and Simulation Group (IGS), University of Innsbruck, Innsbruck, Austria; 2 Interventional Oncology-Microinvasive Therapy (SIP), Department of Radiology, Medical University Innsbruck, Innsbruck, Austria

**Keywords:** Liver segmentation, In the wild, Contrastive, Distillation

## Abstract

**Purpose:**

Automatic liver segmentation is a key component for performing computer-assisted hepatic procedures. The task is challenging due to the high variability in organ appearance, numerous imaging modalities, and limited availability of labels. Moreover, strong generalization performance is required in real-world scenarios. However, existing supervised methods cannot be applied to data not seen during training (i.e. in the wild) because they generalize poorly.

**Methods:**

We propose to distill knowledge from a powerful model with our novel contrastive distillation scheme. We use a pre-trained large neural network to train our smaller model. A key novelty is to map neighboring slices close together in the latent representation, while mapping distant slices far away. Then, we use ground-truth labels to learn a U-Net style upsampling path and recover the segmentation map.

**Results:**

The pipeline is proven to be robust enough to perform state-of-the-art inference on target unseen domains. We carried out an extensive experimental validation using six common abdominal datasets, covering multiple modalities, as well as 18 patient datasets from the Innsbruck University Hospital. A sub-second inference time and a data-efficient training pipeline make it possible to scale our method to real-world conditions.

**Conclusion:**

We propose a novel contrastive distillation scheme for automatic liver segmentation. A limited set of assumptions and superior performance to state-of-the-art techniques make our method a candidate for application to real-world scenarios.

**Supplementary Information:**

The online version contains supplementary material available at 10.1007/s11548-023-02912-3.

## Introduction

**Fig. 1:**
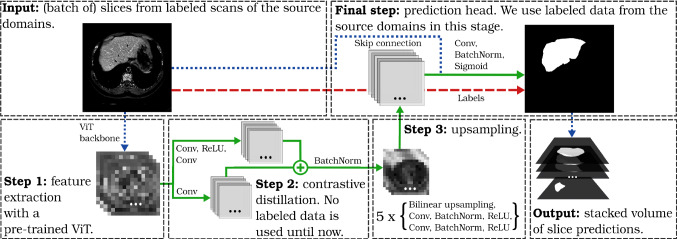
Overview of our training pipeline. It comprises three main processing steps: feature extraction, contrastive distillation, and learnable upsampling. Dots indicate numerous channels (typically hundreds). Learnable steps are shown in green solid lines, non-learnable in blue dotted lines, and label usage is marked with red dashed lines

Medical image segmentation is a key step toward automatic computer-assisted procedures. The high variability of organ appearance, numerous different modalities, the absence of texture contrast, and the limited availability of labels are some of the challenges of segmenting medical scans *in the wild* (i.e. generalizing well to arbitrary real-world datasets not seen during training). Despite efforts in computer vision (CV) and medical image analysis, the task is still considered unsolved. Recently, fully supervised deep learning methods have achieved human-level segmentation performance on synthetic datasets [1–3]. However, their performance degrades rapidly in real-world scenarios [4], where a large distribution shift between training and inference data is often encountered. The issue is more evident in the medical domain, where generalization to real-world clinical settings is hard even for state-of-the-art models [5]. The large shift between the fields hampers the application of common CV solutions in the medical domain. Extensive pre-training on natural images does not always help to transfer knowledge [6]. The discrepancy between the two domains is considerable: ImageNet [7] (a common CV dataset) contains 14.2 million images from 22 thousand classes, while LiTS [8] (a common abdomen tomography dataset) contains 131 volumes with up to 30000 images and two classes. Domain adaptation (DA) techniques assume access to the inputs of the target domain. However, this is not valid in real-world in the wild scenarios, wherefore domain generalization (DG) techniques have been proposed. These either assume access to many datasets (i.e. multi-DG), which is prohibitive in real-world scenarios, or train on a single, heavily augmented dataset (i.e. single-DG), which is not easily scalable in real-world scenarios.

Related to this, in this paper we address the problem of automatic liver segmentation in the wild. Our input data are 3D tomographic scans obtained from medical imaging, which are processed as a set of 2D slices along the axial direction. Our pipeline does not undergo any pre-training or self-supervised phase. We train on a small number of labeled inputs from one or more source domains and perform inference on the unseen target domains. Our method learns to construct a robust latent representation from abdominal scans with a novel contrastive “distillation” scheme. We transfer knowledge from a pre-trained large model to our smaller model under a contrastive framework by imposing that neighboring slices (along the cranio-caudal *z*-axis of the scans) are mapped close together in the latent space, while distant slices are mapped far away, when measured e.g. with cosine similarity. Then, we perform a U-Net-style upsampling to recover the segmentation prediction. An overview of the pipeline can be seen in Fig. 1. Our key contribution is a contrastive sampling strategy suitable for liver segmentation in the wild. Knowledge distillation in our contrastive framework achieves state-of-the-art domain generalization results. We perform an extensive benchmark of our method using six public medical datasets, commonly used in the field, as well as real-world scans from the Innsbruck University Hospital. Finally, we provide ablation studies to justify the design choices of the pipeline.

## Related work

Traditionally, automatic liver segmentation has been tackled via region-growing, rule-based, graph-cut, or statistical-shape-model approaches [9]. Only recently deep learning-based methods have started to surpass traditional techniques [8, 10]. Therefore, in the following, we will focus on recent state-of-the-art for automatic anatomical segmentation with deep learning.

*Domain generalization for medical image segmentation* Recent work reported in [11, 12] attempts to solve the DG problem in medical segmentation via episodic training (the model is trained on train-test splits of a virtual dataset). In particular, Li et al. show superior performance in multi-DG for liver segmentation in computed tomography (CT) data [12]. However, many different labeled domains are required. The generalization to data in the wild is mostly given by the meta-split generation process and the difference across the meta-training domains. The learning objective in both works does not explicitly include the construction of a robust latent representation that can be leveraged across domains. Regarding single-DG methods, the work described in [13] focuses on cross-modality segmentation by augmenting individual domains and capturing domain-specific information to simulate the appearance of unseen domains. The authors achieve great performance in single-DG on multi-organ abdominal tomography segmentation.

*Contrastive learning for medical image segmentation* Pre-training a model on a huge amount of data and fine-tuning it to a few labels is not always useful for medical image segmentation [6]. This is why contrastive learning approaches have been explored. The technique is typically used in low-data regimes to learn powerful representations. The idea is to pull together the feature representations of similar data points (i.e. positive samples), while pulling apart those of dissimilar data (i.e. negative samples). The work described in [14] proposes a contrastive approach based on tomography slices for pre-training their model, focusing on cardiac tomography segmentation. However, as the experimental results show, their solution is suitable only for small organs (w.r.t. a full abdominal scan). Another difficulty is their assumption of perfect alignment between volumes and datasets. The authors of [15] introduce a contrastive distillation loss in order to solve unsupervised semantic segmentation. While we draw on their work to define our contrastive distillation strategy, our pipeline is trained on labeled data from one or more source datasets with the goal of generalization to unseen target datasets. Moreover, our pipeline heavily differs in contrastive sampling strategy and learning objective.

## Method

*Feature extraction*  We draw on the evidence provided in [16] and employ a *frozen* (i.e. gradients do not get back-propagated) self-supervised Vision Transformer (ViT) [17] as feature extractor in the first step of our pipeline. Freezing the backbone is necessary to avoid the risk of overfitting the training data (distillation performance should be domain invariant) and helps reducing the model training time.

*Contrastive distillation* In the second step, we carry out contrastive distillation on the obtained features. Given a feature extractor $\lambda$ and two input images *x*, *y*, the metric $\mathcal {X}$ computes the cosine similarity between the feature tensors $\lambda (x) \in \mathbb {R}^{C \times H \times W}$ and $\lambda (y) \in \mathbb {R}^{C \times I \times J}$, at spatial positions (*h*, *w*) and (*i*, *j*), respectively:

1 $$\begin{aligned} \mathcal {X}_{\scriptscriptstyle hwij}(\lambda , x, y)&= \sum _c \frac{\lambda _{chw} (x) \cdot \lambda _{cij} (y)}{\Vert \lambda _{hw} (x) \Vert \Vert \lambda _{ij} (y) \Vert }, \end{aligned}$$

where *c* is the channel dimension. Based on this, we determine the distillation loss $\mathcal {L}_{cd}$ as a key element of our loss formulation, following [15]. It is imposed on each input slice sample *x* to learn a nonlinear transformation of the preliminary feature tensor:

2 $$\begin{aligned} \mathcal {L}_{cd}(x)&= \mathcal {L}_{cr}(x, x, b_{\text {self}}) + \mathcal {L}_{cr}(x, x^+, b_+) \nonumber \\&\quad +\, \mathcal {L}_{cr}(x, x^-, b_-) \end{aligned}$$

3 $$\begin{aligned} \mathcal {L}_{cr}(x, y, b)&= -\sum _{\scriptscriptstyle hwij} (\mathcal {S}_{\scriptscriptstyle hwij}( \mathcal {X}_{\scriptscriptstyle hwij}(\mathcal {F}, x, y) ) \nonumber \\&\quad -\, b) \max (\mathcal {X}_{\scriptscriptstyle hwij}( \mathcal {C} \circ \mathcal {F}, x, y), 0)\end{aligned}$$

4 $$\begin{aligned} \mathcal {S}_{\scriptscriptstyle hwij}(X)&= X_{\scriptscriptstyle hwij} - \frac{1}{IJ} \sum _{i' j'} X_{\scriptscriptstyle hwi' j'}, \end{aligned}$$

where $\mathcal {S}_{\scriptscriptstyle hwij}$ is the spatial centering operation introduced in [15], $\mathcal {F}$ is the feature extractor, $\mathcal {C}$ is the nonlinear transformation, *b* is a hyper-parameter to prevent collapse. Here, $x^+, x^-$ denote the positive and negative samples, while $b_{\text {self}}, b_+, b_-$ are the specific hyper-parameters for balancing the learning signal. These are set such that at the end of the training the average similarity of features between a slice and itself equals 0.05, between a slice and its positive samples equals 0.0, and between a slice and its negative sample equals $-0.05$, respectively. To evaluate this setting we examined the distribution of feature correspondences between a slice and itself. A bi-modal distribution peaking at alignment (1) and orthogonality (0) of features resulted. This empirically demonstrates the expected clustering of the slices. Finally, we parameterize the nonlinear transformation with 2 convolutional layers and ReLU nonlinearities.

*Contrastive sampling strategy* To work with a wide range of liver shapes and positions, our method takes into account the relative position of a slice in the scan. A sampling strategy with pre-defined thresholds provides false positives and negatives due to the high variance of liver metrics between patients. Therefore, we propose to obtain positives from neighboring slices ($i-1$ and $i+1$) of a given *i*-th slice of a scan *I*, and negatives from the farthest possible one, i.e. $i + \frac{\mid I\mid }{2}$, where $|I |$ is the number of slices in *I*. This contrastive sampling strategy is tailored for large and highly deformable organs such as the liver.

*Learnable upsampling* The third step in the pipeline is the upsampling yielding the segmentation predictions. We follow the traditional U-Net scheme [18], learning five upsampling-then-convolution layers to decode the latent representation and recover spatial information. Finally, we apply a skip connection to the upsampled representation and the original input, and learn three convolutional layers to generate the segmentation prediction. Batch normalization and ReLU activations are used after each convolutional layer, as well as dropout to improve generalization.

*Training objective* The supervised loss objective in the pipeline consists of several components commonly applied in the context of image segmentation: the Focal loss variant of the traditional cross-entropy loss [19], the classic Tversky loss, and the logarithm of IoU (i.e. intersection over union) loss [20]. Additionally, we impose the entropy-based unsupervised loss [21].

**Table 1:** Quantitative comparison between multi-DG methods and our approach. Results of supervised methods are shown in italic, to give an upper bound; training and inference data are different splits of the same dataset. The best statistically significant results are marked in bold font

Inference data	Source training data	Method	DICE	*p*-Value
BTCV	CHAOS,IRCADb,LiTS	[11]	0.863	$5.38 \times 10 ^ {-14}$
		[12]	0.867	$2.57 \times 10 ^ {-13}$
		Ours	$\mathbf {0.929 \pm 0.026}$	–
	*BTCV*	[3]	$0.985$	$2.34 \times 10^{-12}$
CHAOS	BTCV,IRCADb,LiTS	[11]	0.911	$6.31 \times 10 ^ {-7}$
		[12]	0.919	$9.92 \times 10 ^ {-6}$
		Ours	$\mathbf {0.954 \pm 0.026}$	–
	*CHAOS*	[1]	$0.979 \pm 0.003$	$5.23 \times 10 ^{-5}$
LiTS	BTCV,CHAOS,IRCADb	[11]	0.901	$1.72 \times 10 ^ {-39}$
		[12]	0.897	$5.30 \times 10 ^ {-43}$
		Ours	$\mathbf {0.948 \pm 0.028}$	–
	*LiTS*	[2]	*0.942*	*0.125*

The Focal loss helps to speed up the training process, while the classic Tversky is the common loss objective in medical image segmentation. The logarithm of IoU is concerned with optimizing the salient IoU metric, measuring the overlap between two segmentation maps, and the entropy-based unsupervised loss supports regularization of a segmentation map. We have studied the effect of each loss component, summarized below in Table 4.

## Experiments and results

*Experimental setup* We have evaluated and compared our method on the liver segmentation task using specifically prepared medical datasets (BTCV [22], CHAOS [23], IRCADb [24], LiTS [8], ACT-1K [25], and AMOS22^1^), as well as real-world scans from Innsbruck University Hospital (IUH). To support that our method can be successfully applied in the wild, we devised three leave-one-dataset-out cross-validation multi-DG experiments (i.e. training on multiple source datasets and testing on the remaining unseen one, as commonly done in related works [11, 12]). Further, we examined the performance on cross-dataset and cross-modality in a single-DG study. Finally, we qualitatively evaluated the results on real-world clinical scans.

For training, we employ the RAdam optimizer [26] that facilitates faster learning with an adaptive learning rate mechanism (batch size 8, weight decay $10^{-5}$). The initial learning rate set to $10^{-4}$; it is reduced when there is no metric improvement on the training test set after 10 epochs. We found that 150 epochs are sufficient for convergence based on the DICE metric evaluated on the source data test set. As test set, we employed 5 scans of each training dataset. The training was done with a ViT-base/8 backbone, on Linux Ubuntu with 16 GB RAM, 8 $\times$ Intel Core i7-9700K CPU @ 3.60GHz, 8 GB NVIDIA GeForce RTX 3080 Ti. PyTorch 1.10 and TorchIO [27] were employed to implement our pipeline. Further details on the pipeline are compiled in supplementary material.

**Fig. 2:**
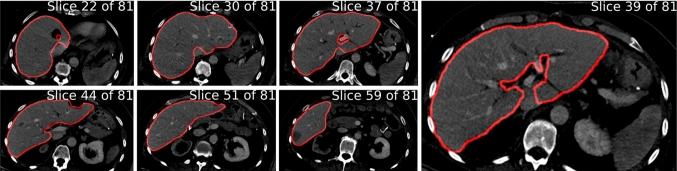
Liver segmentation predicted by our method on a case in portal venous phase from the IUH

*Metrics* For comparison we use the DICE metric, which is the only metric consistently reported in related work. It estimates similarity of two samples via the number of pixels in common. Statistical significance of our results is examined via *p*-values from paired *t*-tests on directly compared DICE coefficients (assuming normality of results). Any standard deviations are computed across scans. In the following tables, the best statistically significant results (*p*-value $< 0.05$) are marked in bold font. Note that this only takes into account the other unsupervised methods; metrics of fully supervised methods are just provided to give an upper bound on the performance. Fully supervised methods have access to the inference data distribution, rendering a direct comparison with unsupervised, in the wild methods unfair. It has to be noted that in some cases it was not possible to obtain the implementations from other authors for comparisons, wherefore we were only able to compare to their published results. To this end, we sampled from a normal distribution centered at the mean reported in the respective publications, assuming 0 standard deviation.

*Evaluation on widely available medical datasets* First, we have carried out leave-one-domain-out experiments, in multi-DG settings. The results are reported in Table 1. For training, different combinations of the BTCV, CHAOS, IRCADb, and LiTS datasets were used, excluding one; and inference was performed on the remaining dataset. We compare to the performance of the unsupervised approaches in [11, 12]. Methods [1–3] are *supervised* state-of-the-art in the respective inference domain, giving an upper bound.

Next, we compared to the state-of-the-art single-DG technique reported in [13]. Following their work, we trained on the CT data of the BTCV dataset and inferred on the MR T2-SPIR data of the CHAOS dataset. We obtained with our method a DICE metric of $0.815 \pm 0.087$, compared to 0.673 for [13], with a statistically significant *p*-value of $8.39 \times 10 ^ {-7}$.

Further, we performed inference on the scans from the AMOS22 challenge (using our model trained on the BTCV, CHAOS, IRCADb datasets), and obtained a DICE score of $0.918 \pm 0.066$, compared to 0.981 for the fully supervised method described in Isensee et al.^2^ (*p*-value $5.02 \times 10^{-30}$). To the best of our knowledge, these are the first in the wild results on AMOS22 challenge data.

In addition to the DICE results reported above, we also examined two further salients: average symmetric surface distance (ASSD) and maximum symmetric surface distance (MSSD). The former computes the average distance between the predicted and the ground truth surface, while the latter considers only the maximum distance. These values are often not reported in related work, but we consider them useful from a clinical perspective. They evaluate the error on the real-world-dimensions of a scan, not just on pixels. The ASSD metric was under 1.3 mm in each experiment ($1.057 \pm 0.537$ mm, $0.757 \pm 0.380$ mm, and $1.299 \pm 1.829$ mm, for BTCV, CHAOS, and LiTS, respectively). The MSSD metric was found to be smaller than 5 cm ($4.108 \pm 2.93$ cm, $3.09 \pm 2.40$ cm, and $4.62 \pm 5.20$ cm, for BTCV, CHAOS, and LiTS, respectively).

*Evaluation on clinical scans in the wild* Further, we performed inference on everyday patient CT scans of 18, obtained in preparation for stereotactic radiofrequency ablation (SRFA) interventions [28]. An overview of one segmented case can be seen in Fig. 2; more visual results and comparisons with commercial systems are shown in supplementary material. Our segmentations were found to be reasonable and useful for their clinical practice by our collaborating radiological experts. A more quantitative evaluation will be carried out in the future.

## Ablation studies and discussion

*Ablation studies* We conducted several ablation studies, on training data, pipeline, contrastive distillation loss, objective loss, and contrastive sampling strategy. For this, we examined inference on the BTCV dataset, as it showed the lowest performance (see Table 1). We report *p*-values of paired *t*-tests with respect to DICE metrics, comparing against our pipeline with a ViT-small/16 feature extractor and default parameters as baseline.

**Table 2:** Effect of the amount of training data in source domains on the DICE performance in the target domain (BCTV). Results of baseline method are shown in italic. The best statistically significant results are marked in bold font

CHAOS	IRCADb	LiTS	Method	DICE	*p*-Value
5	5	5	Ours	$0.895 \pm 0.038$	0.298
10	10	10		$0.903 \pm 0.033$	0.875
15	15	15		$0.904 \pm 0.030$	*Baseline*
14	14	92		$\mathbf {0.915 \pm 0.029}$	0.168
14	14	92	[12]	0.867	$2.57 \times 10 ^ {-13}$

First, we studied the effect of the amount of training data in the source domains on the DICE performance in the target domain (BCTV). Results are reported in Table 2. Comparisons were done with multi-DG state-of-the-art [12]. We found that using as little as 5 scans in each source training domain was sufficient to reach competitive performance. This is important for work in the wild, since learning has to be done efficiently in low-data regimes. Moreover, increasing the amount of training data in general seemed to increase the performance, but without statistical significance.

**Table 3:** Ablation studies on the contrastive sampling strategy. Results of baseline method are shown in italic

Positive samples	Negative samples	DICE	*p*-Value
$i-1$-th, $i+1$-th slices	$i + \frac{\mid I \mid }{2}$-th	$\mathbf { 0.904 \pm 0.030 }$	*Baseline*
$i-2$-th and $i+2$-th slices	$i + \frac{\mid I \mid }{2}$-th	$0.892 \pm 0.038$	0.168
$i-1$-th and $i+1$-th slices	$i + \frac{\mid I \mid }{2}$-th, $i + \frac{\mid I \mid }{2} + 1$-th	$0.888 \pm 0.042$	$8.92\times 10 ^{-2}$

**Table 4:** Ablation studies grouped by data, pipeline module and loss function examined

Trainingdata	*RS*	Backbone	Loss ablated	Loss range	DICE	*p*-Value
ACT-1K	42	ViT-small/16			$0.902 \pm 0.049$	0.866
CHAOS IRCADb LiTS	42	ViT-small/16			$0.904 \pm 0.030$	*Baseline*
	41				$0.903 \pm 0.035$	0.849
	43				$0.906 \pm 0.031$	0.838
	44				$0.905 \pm 0.029$	0.892
	42	ViT-small/8			$0.921 \pm 0.035$	0.048
		ViT-base/16			$0.913 \pm 0.031$	0.251
		ViT-base/8			$\mathbf {0.929 \pm 0.026}$	$1.67\times 10 ^{-3}$
	42	ViT-small/16	$\mathcal {L}_{cd}$	$[-1, 0]$	$0.876 \pm 0.030$	$1.42\times 10 ^{-2}$
			Focal	$[0, + \infty [$	$0.902 \pm 0.032$	0.825
			Tversky	[0, 1]	$0.900 \pm 0.031$	0.590
			$\log$ IoU	$[0, + \infty [$	$0.912 \pm 0.028$	0.321
			Entropy	[0, 0.02[	$0.901 \pm 0.030$	0.692

The target inference dataset is BTCV. *RS* denotes the randomization seed, encoding different splits of training data

Next, we examined the influence of our sampling strategy for positive and negative samples. Different approaches for obtaining these samples were compared. Results are compiled in Table 3. Input is the *i*-th slice of a scan *I*, where $|I |$ is again the number of slices in *I*. The target inference dataset is again BTCV. Sampling positive samples from neighboring slices, and negative samples from the farthest possible yielded overall the best performance.

Finally, we studied the influence of training data, different data splits, backbone sizes, and loss functions. The results are compiled in Table 4. As can be seen, the choice of training data splits (encoded by the randomization seed *RS*) or the usage of a different dataset (ACT-1K) did not influence the inference performance. Further, as expected, using more powerful backbones (ViT with patch resolution of 8) improved the performance. Regarding the loss functions, dropping the contrastive distillation (see row 9) yielded a significant drop in performance. We also note that omitting the $\log$ IoU loss from the learning objective achieved a higher, but not statistically significant, DICE metric. However, since the loss optimizes the number of false positives better than the Tversky loss, it is part of our learning objective.

*Comparison with state-of-the-art and limitations* Overall, the experimental results show that our method surpasses the performance of state-of-the-art multi-DG methods with statistical significance. Our method also performed better than fully supervised methods in the case of LiTS inference. When comparing with single-DG methods, there is a significant performance improvement over the state-of-the-art [13]. Finally, our method was capable of segmenting the liver in challenging conditions in the wild, even in presence of SRFA needles (see a comparison with commercial systems in supplementary material).

As a limitation, our method has difficulties in predicting the segmentation map at the bottom of the liver. We hypothesize the reason for this is the downsampling in the pre-processing steps, since it may misalign the learned convolution kernels with the tiny liver part. This could be solved by using higher-resolution slices. As evidenced by evaluations on the clinical scans, the scans in hepatic arterial phase are easier to segment than the other phases; the scans with no contrast enhancement are the most difficult to segment.

## Conclusions and future work

Our novel contrastive distillation scheme showed great potential for automatic liver segmentation in the wild. Thorough ablation studies and empirical results on common test datasets and real-world scans support these findings. Our next steps aim at multi-modal multi-organ segmentation, as this is critical for computer-assisted hepatic procedures. Using powerful backbone architectures has a beneficial effect on performance, however, there is a clear discrepancy between what can be achieved in the natural imaging domain and the medical imaging domain. We will explore cross-domain knowledge transfer that was recently reported in other domains.^3^

## Supplementary Information

Below is the link to the electronic supplementary material.

Supplementary file 1 (pdf 2438 KB)

Supplementary file 2 (mp4 1112 KB)

Supplementary file 3 (mp4 1446 KB)

## Data Availability

The publicly available datasets BTCV, CHAOS, D-IRCADb-01, LiTS, ACT-1K, and AMOS22 [8, 22–25] were used, containing 30, 20, 20, 131, 1000, 200 patient scans each. Patients at IUH gave informed consent for anonymous use of their data. Code and pre-trained models can be found at https://git.uibk.ac.at/informatik/igs/open/zigpub.

## Footnotes

1 Ji et al., AMOS: A Large-Scale Abdominal Multi-Organ Benchmark for Versatile Medical Image Segmentation, NeurIPS Track Datasets and Benchmarks (2022).

2 Isensee et al., Extending nnU-Net is all you need, arXiv pre-print (2022).

3 Kaplan et al., Scaling Laws for Neural Language Models, arXiv pre-print (2020).
