# Supplementary material for: Towards liver segmentation in the wild via contrastive distillation
Source: Int J Comput Assist Radiol Surg. 2023 May 5;18(7):1143–9. doi: 10.1007/s11548-023-02912-3 (PMC10329587; doi:10.1007/s11548-023-02912-3)
Supplement: Supplementary file 1 — (pdf 2438 KB) [file 11548_2023_2912_MOESM1_ESM.pdf]

Appendix A Supplementary Information

A.1 Experiments

Data pre-processing We thresholded the Hounsfield scale (normalized scale for CT scans) to $[0, 500]$ Hounsfield units (HU) for CT data and $[0, 1.5]$ for MR T2-SPIR data. We removed outliers based on the upper and lower 2 percentiles, normalized to $[0, 1]$, and standardized to zero-mean-unit-variance each scan. In the single-DG experiments, we followed related works and pre-processed the CT data by thresholding the inputs at 125 HU. To extract the preliminary features with the pre-trained backbone, we resized each slice to 224×224.

Labeled data To promote dataset consistency (only CHAOS dataset contains healthy liver), pixels belonging to liver tumors are considered part of the liver in the experiments. The performance is neither degraded nor improved by this label treatment: the p-value of the paired t-test on the DICE metric is 0.910.

Training During training, the data has been augmented with standard techniques: elastic deformation, blurring, noise, and gamma histogram transformation. Training time varies according to which backbone is used: with a ViT-small/16 backbone and a dataset of ≈ 10000 slices the training procedure converges in ≈ 6 hours.

Inference time Thanks also to the relatively small number of parameters of our model ($2.5 \cdot 10^5$, compared to $3 \cdot 10^6$ of the traditional U-Net architecture [18]) the inference time per scan (126 slices on average, with resolution 224×224) is 0.591 ± 0.082 seconds with a ViT-small/16 backbone.

Prediction post-processing We binarized the predicted segmentation masks based on the 0.5 threshold, like in related works. In the evaluation phase, we resized each scan slice to 128×128, following related works. In the single-DG experiments, we resized each scan slice to 192×192, like in related works.

Model architecture and hyper-parameters Table A1 lists the hyper-parameter values not listed in the main paper.

Method comparisons In the multi-DG experiments, we did not perform inference on the 3D-IRCADb-01 dataset because the LiTS dataset already includes it.

Data availability The study used the publicly available datasets BTCV, CHAOS ([22], https://chaos.grand-challenge.org), 3D-IRCADb-01 ([23], https://www.ircad.fr/research/data-sets/liver-segmentation-3d-ircadb-01/),

Table A1: Hyper-parameter listing.

scope	parameter	value
elastic augmentation	p	0.9
	σ	3.5
	number of points	8
model	latent code dimensions	70
	p backbone	0.1
	non-linear transformation kernel size	1×1
	non-linear transformation dropout	0.1
	upsampling channels	$(70), 40, 20, 10, 5$
	skip connection kernel size	3×3
	prediction kernel size	3×3
	prediction activation	sigmoid
	p dropout	0.3
Focal loss	α	0.5
	γ	3.0
contrastive distillation loss	b_-	0.6
	b_{self}	0.55
	b_+	0.5
noise augmentation	p	0.6
	μ	input mean
	σ	0.15
blur augmentation	p	0.6
	σ	0.7
gamma augmentation	p	0.6
	log	$[0.1, 0.9]$
	σ	0.15
dropout augmentation	p	0.2

LiTS ([8], https://competitions.codalab.org/competitions/17094), AbdomenCT-1K ([24], https://github.com/JunMa11/AbdomenCT-1K) and AMOS22 (https://amos22.grand-challenge.org/) datasets.

A.2 Inference on Innsbruck University Hospital CT scans

Patient cohort We sampled 18 CT scans from 18 random patients (10 males, 8 females, aged 65.6 ± 12.3 year old) that were treated by SRFA at the Innsbruck University Hospital [27]. The SRFA procedure entails thermal ablation of liver tumors with a multiple-needle stereotactic approach. A precise 3D planning on multi-modal pre-procedural scans and the insertion of coaxial needles in the patient are the first two steps of the procedure. Needle placement is verified via fusion of pre-procedural and intra-procedural control scans. Next, alternating current passes through the ablation probes and thermal energy is transmitted to the target tissue. Once the target tissue temperature is reached (e.g. 60 Celsius degree) irreversible destruction of the tumor tissue nearby the needle is achieved. The abundance of tumoral areas,

residues of ablation zones, and the presence of ablation probes are some of the difficulties in performing liver segmentation on SRFA scans. Moreover, intraprocedural CT scans feature various sorts of artifacts due to the uncontrolled imaging acquisition environment (varying dose, type of contrast agent, and patient position).

Comparison to commercial systems Two of the most successful commercial systems for automatic liver analysis are Siemens *syngo*.via and Ablation-fit. Since Ablation-fit requires two different contrast-enhanced liver phases, in the following we report comparison just with the Siemens system. We used the automatic "CT Liver Analysis" program of Siemens *syngo*.via to open the patient scans. It takes 13.966 ± 2.377 seconds to perform the liver segmentation using a Windows 10 workstation with 32 GB RAM, 8 $\times$ Intel Core i7-10700K CPU @ 2.90GHz, 2 GB NVIDIA GeForce GT 1030. Siemens *syngo*.via exports segmentation results as dotted contours, so we performed dilation and skeletonization to recover a continuous contour.

For space reasons, only three interesting cases are shown: we chose them because they are in hepatic arterial phase and portal venous phase, commonly used for planning and verification of the procedures. An expert interventional radiologist confirmed that our predictions are at least of comparable quality as the results from Siemens *syngo*.via. Refer to the supplementary videos showing two other cases collected in hepatic arterial phase and portal venous phase for a more in-depth analysis.

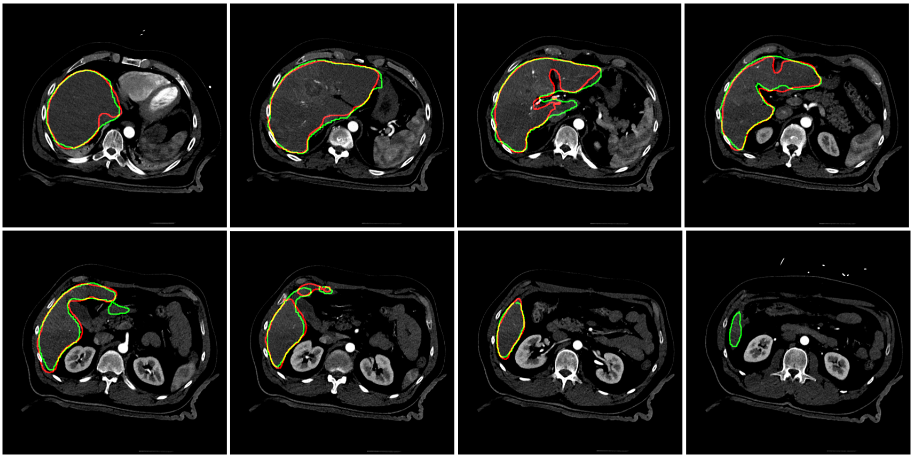

Figure A1: Liver segmentation prediction on a planning CT scan (100 mL Visipaque 320, arterial phase) in a 45 year old male patient. The prediction of our method is shown in red and the result using the Siemens *syngo*.via software is displayed in green. The Siemens system mistakes the inferior vena cava for liver tissue. Our method cannot segment the liver in the inferior part.

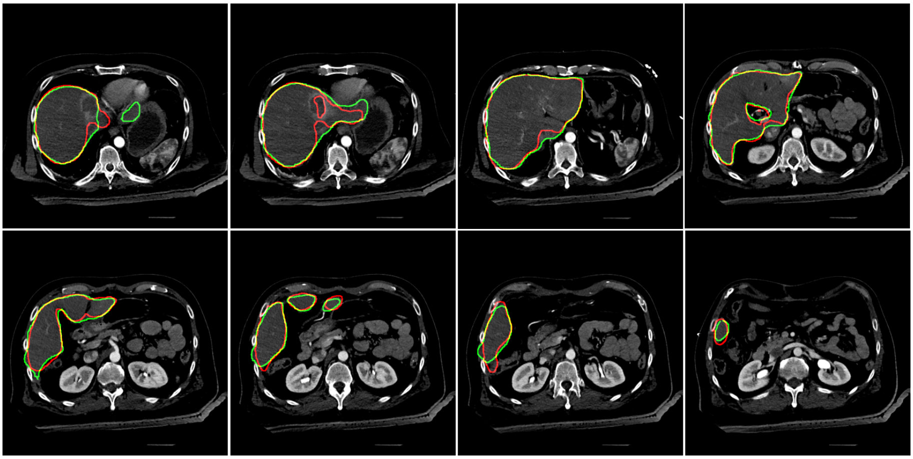

Figure A2: Liver segmentation prediction on a planning CT scan (74 mL Ultravist 370, arterial phase) in a 45 year old male patient. The prediction of our method is shown in red and the result using the Siemens *syngo*.via software is displayed in green. The Siemens system includes skin in the liver segmentation (second row). Note that our method successfully avoids segmenting the SRFA ablation zone (second slice).

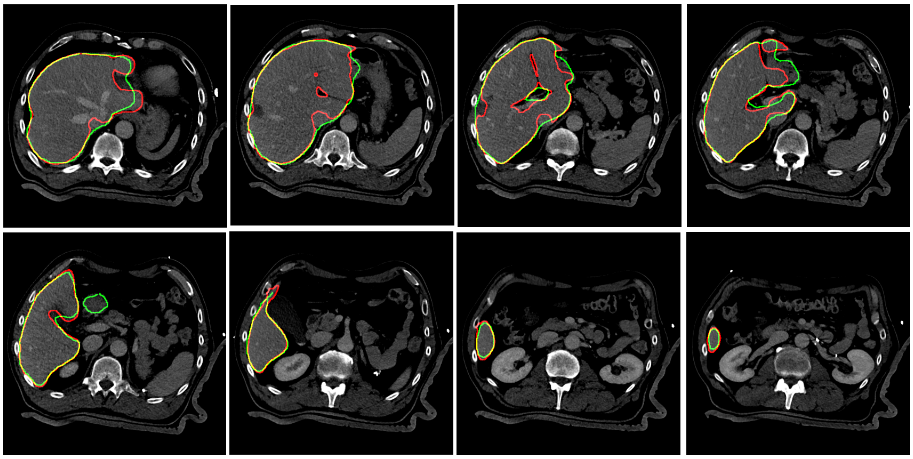

Figure A3: Liver segmentation prediction on a planning CT scan (100 mL of Jopamiro 300, portal venous phase) in a 61 year old male patient. The prediction of our method is shown in red and the result using the Siemens *syngo*.via software is displayed in green. The Siemens system mistakes the inferior vena cava and part of the gallbladder for liver tissue (second row on the left), while our method shows some inaccuracies in the lower part of the liver close to the ribs.
